# The Role of PROX1 in Neoplasia: A Key Player Often Overlooked

**DOI:** 10.3390/diagnostics12071624

**Published:** 2022-07-04

**Authors:** Evangelia Ntikoudi, Alexandros Pergaris, Stylianos Kykalos, Ekaterini Politi, Stamatios Theocharis

**Affiliations:** 1 First Department of Pathology, National and Kapodistrian University of Athens, 11527 Athens, Greece; dikoudi@yahoo.com (E.N.); alexperg@yahoo.com (A.P.); 2 Second Propaedeutic Department of Surgery, National and Kapodistrian University of Athens, General Hospital of Athens “Laiko”, Agiou Thoma 17, 11527 Athens, Greece; kykalos@gmail.com; 3 Department of Cytopathology, Aretaieion Hospital, Medical School, National and Kapodistrian University of Athens, 11528 Athens, Greece; ekpoliti@med.uoa.gr

**Keywords:** *PROX1*, lymphangiogenesis, biomarkers, cancer, diagnosis, prognosis, therapy

## Abstract

The human *PROX1* gene (*Prospero homeobox gene 1*) is a member of the homeobox transcription factor family. PROX1 plays a key role in the development of the lymphatic system and is primarily used as a lymphatic vessel marker. However, as the accumulating evidence indicates that PROX1 is also implicated in the tumorigenesis of various cancer types, the scientific community has attempted to elucidate its complicated function in neoplasia pathogenesis, as well as its utility in cancer diagnosis, prognosis, and therapy. PROX1 has been shown to participate in the complex molecular mechanisms affecting tumorigenesis and has been associated with a plethora of clinicopathological parameters, including tumor stage and patients’ overall survival. Depending on the specific organ affected, PROX1 has exhibited both tumor-promoting and tumor-suppressing properties, with its inhibition and reactivation representing possible novel therapeutic interventions, respectively. Moreover, researchers have reported PROX1 as a useful tool in the fields of diagnosis and prognosis assessment. The current study aims to summarize and present the existing data that render PROX1 a novel and useful diagnostic and prognostic biomarker, as well as a possible therapeutic target.

## 1. Introduction

Neoplastic disease remains one of the leading causes of morbidity and death worldwide, with its heavy clinical and social impact posing a major challenge for the medical community. As our accumulating knowledge sheds light on the molecular pathways implicated in cancer pathogenesis, the development of novel, specific therapies targeting key oncogenic mechanisms is of the utmost importance. Tumor progression and metastasis are strongly directed by angiogenesis and lymphangiogenesis. Numerous transcription factors are involved in the complex process of cancer biology, leading either to the promotion or the inhibition of carcinogenesis.

The human *PROX1* gene (Prospero homeobox gene 1) is a member of the homeobox transcription factor family. Members of this family contain a homeobox domain that consists of a 60-amino acid helix-turn-helix structure that binds DNA and RNA. The *PROX1* gene is related to the *Drosophila prospero* gene, encoding a nuclear transcription factor. The PROX1 protein is mainly expressed in endothelial cells that give rise to the lymphatic system, serving as a marker for mammalian lymphatic endothelial cells. It is also involved in the development of various organs, such as the heart, retina, liver, pancreas, and central nervous system [1].

PROX1 mediates its lymphangiogenic action by regulating the expression of various lymphatic transcription factors, such as vascular endothelial growth factors (VEGFs) or the lymphatic vessel endothelial hyaluronan receptor-1 (LYVE-1). Interestingly, PROX1 is believed to act as an additional angiogenic factor, mostly via the activation of the vascular endothelial growth factor (VEGF) receptor-3 (VEGFR-3). The blood, vascular, and lymphatic systems have a common embryogenic origin. During development, PROX1, among other factors, strongly directs their further development toward lymphatic endothelial cell differentiation. It is noteworthy that the overexpression of PROX1 in blood endothelial cells will transform them into lymphatic endothelial cells [2]. Moreover, the cell identity of the lymphatic endothelial system is reversible and is highly dependent on PROX1 activity. That means that in cases of PROX1 silencing, a reverse differentiation of the lymphatic endothelial cells into blood endothelial cells may occur [3].

PROX1 expression is considered both necessary and sufficient to turn the blood endothelial cell phenotype toward the lymphatic endothelial cell phenotype via various and complex molecular mechanisms, besides the regulation of LYVE-1. LYVE-1-deficient mice were able to develop a lymph vasculature that was normal in both architecture and function [4], in contrast to the PROX-1-deficient embryos, which were devoid of lymph vessels, leading to embryonic lethality [5,6]. PROX1 expression correlates with the downregulation of blood vessel marker expression, such as in endoglin, laminin, intercellular adhesion molecule-1 (ICAM-1), low-density lipoprotein receptor, neuropilin-1, thrombomodulin, VEGFR-2, and CD34, and the upregulation of expression of genes characteristic of lymph vasculature, including podoplanin, VEGFR-3, and integrin α9, with no effect being noted regarding the expression of the pan-endothelial marker, CD31 [5,7,8]. PROX1 further exerts its lymphangiogenic action by upregulating fibroblast growth factor receptor 3 (FGFR3), a tyrosine kinase receptor also implicated in lymphangiogenesis [9]. Moreover, PROX1 expression is linked to certain morphological changes in endothelial cells, the promotion of motility, and the blockage of sheet formation, all of which constitute key procedures of lymphangiogenesis during embryogenesis [7].

The complex molecular pathways of tumor angiogenesis, as well as its role in carcinogenesis, have been the subject of extensive research. Tumors are unable to develop beyond a few millimeters in diameter in the absence of neovascularization. The numerous subtypes of vascular endothelial growth factors (VEGFs) exhibit their action by stimulating specific receptors (the so-called VEGFRs). VEGF-A and VEGF-B are considered key mediators through their binding to VEGFR-1 and -2, stimulating the proliferation and migration of endothelial cells, ultimately leading to angiogenesis [10]. Sprouting vessels provide tumors with oxygen and the nutrients essential for their growth, as well as with a new pathway for invasion and distant spread.

While tumor neovascularization has been thoroughly studied, key information concerning tumor lymphangiogenesis remains as yet unexplored. It is established that lymph vessel formation also contributes to cancer pathogenesis, serving as a route for cancer cell dissemination. In addition, tumor lymph vessels exhibit abnormal functions, such as increased permeability and the inability to remove interstitial fluid, leading to edema, as well as immunomodulating properties that suppress the proper immune response.

VEGF-C and VEGF-D, acting through VEGFR-2 and VEGFR-3, have been reported as central molecules driving lymphangiogenesis, invoking the proliferation, survival, and migration of lymphatic endothelial cells. As previously mentioned, VEGFR-3, the specific receptor of VEGF-C, represents a direct target of PROX1, with known bidirectional feedback regulation [11]. Moreover, it is observed that vascular and lymphatic endothelial cells exhibit common receptors, such as VEGFR-2 and VEGFR-3, suggesting that angiogenesis and lymphangiogenesis are procedures that are far from independent from each other, as they share common molecular pathways [12]. However, it should be highlighted that mature VEGFs (including VEGF-C) are finally produced after several proteolytic cleavages of their inactive propeptides. Various processed forms of VEGFs show an altered affinity for several receptors. This condition could partially explain the mixed effects of VEGF-C, which, depending on the processing, are preferentially lymphangiogenic (binding to the lymphendothelial-specific receptor, VEGFR-3/ flt4) or even angiogenic (binding to the angiogenic receptor, VEGFR-2/ flk1) [13].

The application of anti-VEGF medication in cancer patients’ treatment regimens exhibited limited results. This can be attributed to the fact that, apart from VEGF, a plethora of biomolecules has been implicated in the complex pathways of lymph- and angiogenesis. Further research focusing on these mechanisms is required, so that novel, specialized therapeutic strategies targeting the formation of tumor lymph vessels can be developed.

PROX1 directly interacts with histone acetyltransferase p300 (p300 HAT or EP300), an enzyme that regulates gene transcription through chromatin remodeling. Although the functional relationship is not clear, EP300 seems to be less effective in cases of co-activation with PROX1 [14]. EP300 is speculated to regulate cell growth and further differentiation, inhibiting cancerous transformation [15]. Moreover, EP300 functions as a co-activator of hypoxia-inducible factor 1 alpha (HIF1A), leading to the stimulation of hypoxia-induced genes, such as VEGF.

**Figure 1:**
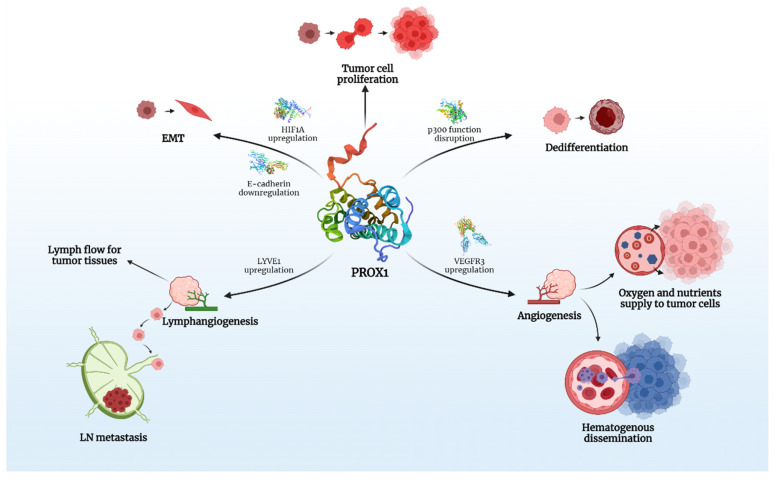
The key roles of PROX1 in carcinogenesis. Created with BioRender.com.

PROX1 is expressed in various human cancers and is speculated to play a crucial role in carcinogenesis and tumor spread (Figure 1).

Taking into account that PROX1 is associated with modifications in the expression of over 150 genes in vitro, its effects on the behavior of various tumors is multifactorial and it is, therefore, able to act as either a promoter or inhibitor of carcinogenesis. There is evidence that PROX1, besides lymph-/angio-genesis, directly interferes with many intracellular signaling pathways, responsible for proliferation and epithelial-mesenchymal transition (EMT) of cancer cells, a process that clearly defines cancer behavior and metastatic potential.

The aim of the present study is to present and summarize existing data concerning PROX1 expression in various cancer types, exploring its role in tumorigenesis. Moreover, the prognostic significance of PROX1 will be elucidated, and its utility as a possible novel therapeutic target will also be discussed.

We conducted an extensive search of the electronic database, Medline, using the keywords PROX1, tumor, pathogenesis, cancer, and prognosis. Inclusion criteria were adult, >18 years old, human, and cancer specimens. Exclusion criteria were case reports, animal studies, cell line studies, congress abstracts, articles not referring to human cancer, and articles not written in English.

## 2. PROX1 Expression in Cancer

### 2.1. Central Nervous System (CNS) Tumors

PROX1, along with other lymphatic markers (LYVE-1 and podoplanin), have been assessed among primary CNS lymphomas (PCNSL), diffuse large B-cell lymphomas (DLBCL), and primary glioblastoma multiforme (GBM) WHO Grade IV, in order to explain the metastatic behavior of the aforementioned CNS tumors [16]. Additionally, there is experimental evidence that VEGF-C-driven lymphatic drainage enables the immunosurveillance of brain tumors [17]. A lack of PROX1 from the PCNSL and GBM samples indicated indirectly the absence of the lymphatic vessels and showed no connection to the lymphatic system. This could explain the topographical restriction and the rarity of metastasis of these tumors. Conversely, dural and meningeal DLBCL showed the positive expression of PROX1, podoplanin, and LYVE-1 in areas where the tumors had invaded the fibrous tissue of the dura. Another study concerning CNS tumors evaluated PROX1 expression in astrocytic gliomas of different histologic grades and revealed a positive correlation with increased tumor grade [18]. High-grade gliomas (III and IV) showed strong positivity for PROX1, while grade II and I tumors or non-neoplastic brain tissue expressed significantly lower PROX1 levels. The increased expression of PROX1 in high-grade tumors could be attributed to an increased number of multipotent progenitors and undifferentiated cells; PROX1 could represent a progenitor cell marker. Thus, it could serve as a tool in the diagnosis of high-grade tumors and aid histological discrimination between Grade II tumors and inflammatory/reactive lesions.

Since Grade II gliomas exhibit a highly variable course and can transform into a more malignant phenotype at any time, the same group of researchers conducted another study in order to evaluate PROX1 as a predictive and prognostic marker in Grade-II gliomas [19]. High PROX1 expression (>30% of the tumor cells) correlated with shorter overall survival (OS). In multivariate analysis, PROX1 expression was identified as an independent prognostic factor for survival.

### 2.2. Oral Cancer

The presence or absence of tumor-infiltrated regional lymph nodes (LNs) is of great significance for the management and prognosis of oral cancer patients. The hallmark of regional lymph node metastasis is the identification of the sentinel lymph node (SLN), which is the first draining lymph node from the tumor. In an effort to elucidate the pathogenesis of regional LN metastasis, Ishii et al. focused on investigating the hypothesis that the primary tumor induces lymphangiogenesis at the SLN before metastasis occurs. They estimated the mRNA expression levels of the lymphatic markers VEGFR-3, PROX1, and LYVE-1 in metastasis-negative SLNs obtained from patients with operable oral squamous cell carcinoma (OSCC), and observed a significant positive correlation between the expression levels of the three lymphatic-specific markers within SLNs from the OSCC patients, suggesting that lymphangiogenesis, probably induced by the primary tumor, does occur in SLNs before the presence of metastases [20].

The expression of PROX1 along with the Forkhead box (FOX) C2, two factors that are involved in angio- and lymphangiogenesis, have been measured in another cohort of OSCC patients [21]. PROX1 was positive in the majority of the nodal metastasis-positive cases and was associated with T classification, clinical stage, and lymphatic vessel density (LVD). PROX1 and FOXC2 expression were associated with poor outcomes in OSCCs. However, multivariate analysis demonstrated only PROX1 expression as a negative prognostic factor for disease-free survival (DFS). The opposite results regarding the prognostic value of PROX1 in OSCC were reported in a study by Rodrigues et al. More specifically, PROX1 expression levels were significantly higher within non-neoplasmatic margins, compared to OSCC, and the expression status of PROX1 showed no significant correlation with any clinicopathological variables. Additionally, cases with positive *PROX1* gene amplification had better survival rates compared with negative cases, suggesting that PROX1 silencing presages an unfavorable prognosis [22].

### 2.3. Thyroid Cancer

There is evidence that PROX1 is consistently downregulated by more than 2-fold in various thyroid cancers, compared to normal tissues. Additionally, in vitro and animal experiments indicate that the downregulation and inactivation of PROX1 might play a significant role in thyroid cancer initiation and development [23]. Moreover, induced *PROX1* gene reactivation in advanced stages of thyroid cancer might represent a novel therapeutic strategy in terms of the inhibition of disease progression.

Whether PROX1, among other vascular factors, enhances thyroid cancer dissemination through the regulation of angiogenesis was the subject of another study. The expression levels of PROX1 and the pro-angiogenic factor fibroblast growth factor (FGF)-2 were measured in paired samples of follicular thyroid cancer and normal tissues. PROX1 expression levels were upregulated in stages I and II and decreased in stages III and IV, while FGF2 levels showed a consistent inverse expression pattern. Additionally, patients with higher PROX1 expression exhibited worse OS. The interdependence between PROX1 and FGF2 was linked to thyroid cancer progression and correlated with the cancer stage and OS. Additional analysis of the extracted data from the GEPIA (gene expression profiling interactive analysis) database [24] led to similar findings, in terms of the association between the PROX1 and FGF2 expression profiles, in a cohort of papillary thyroid carcinoma patients [25].

The data concerning the expression and clinical significance of PROX1 in CNS, OSC, and thyroid cancer are presented in Table 1.

### 2.4. Lung Cancer

Studies investigating the role of PROX1 in lung cancer pathogenesis are relatively sparse.

Regarding small-cell lung cancer (SCLC), Zhu et al. conducted a study using cell lines, showing the oncogenic role of PROX1 in this context. PROX1 was highly expressed in SCLC cell lines, while its knock-down (via a constructed shRNA-lentivirus) significantly reduced the cancer cell proliferation rate [26].

Kowalczuk et al. studied the mRNA levels of molecules that induce lymphangiogenesis in paired surgical specimens obtained from patients with non-small cell lung cancer (NSCLC) at stages I–IIIA. Among the 15 genes analyzed, PROX1, along with podoplanin (PDPN), neuropilin (NRP)-2, and VEGF-A, showed similar expression levels between tumor and normal tissues. When correlated with clinicopathological variables, PROXl levels were only associated with the histological type since they were downregulated in squamous cell lung carcinoma but not in non-squamous tumors. In terms of OS, PROX1 failed to prove itself as a prognostic factor [27].

### 2.5. Breast Cancer

Five studies exist exploring the role of PROX1 in the development and spread of breast cancer.

Van der Auwera et al. investigated whether and to what extent angiogenesis and lymphangiogenesis contribute to the early growth and dissemination of inflammatory breast cancer, a rare breast cancer type with a very aggressive clinical course and poor prognosis. The researchers measured the gene expression of tumor angiogenesis and lymphangiogenesis factors in tumor specimens of inflammatory breast cancer and in control non–stage-matched breast cancer patients [28]. The results showed significantly higher gene expression levels of the lymphangiogenesis-specific factors (VEGF-C, VEGF-D, Flt-4, PROX1, Lyve-1) compared to controls. Furthermore, the inflammatory breast cancer tissues showed higher lymphatic endothelial cell proliferation, compared to non-inflammatory breast cancer. These findings verify the presence of lymphangiogenesis in inflammatory breast cancer and indicate the factors inducing the process in terms of possible therapeutic targets.

In the study by Frewer et al., the levels of interleukin (IL)-24, a cytokine with potential tumor-suppressive activity, its receptor IL-22R, and the levels of lymphangiogenic factors, such as podoplanin, PROX1, and LYVE-1, were evaluated in breast cancer tissue samples. IL-24 and IL-22R were reduced and correlated inversely to the elevated levels of lymphangiogenic markers, suggesting that the lack of IL-24 could enhance lymphangiogenesis, tumor growth, and dissemination in breast cancer. Inversely, the restoration of IL-24 levels could represent an innovative targeted therapy [29].

There is also evidence that primary breast tumors induce lymphangiogenesis via VEGF-C SLN before metastasis occurs. More specifically, Zhao et al. demonstrated that in cases of high VEGF-C-expressing tumors, PROX1 (among other lymphatic-specific markers) was overexpressed in uninvolved sentinel LNs in patients with breast cancer, compared to control LNs (*p* < 0.05) [30].

In contrast to the aforementioned study, Agarwal et al. established via examination of the expression of three lymphatic-specific markers (D240, podoplanin, and PROX1) in breast cancer tissues that, unlike angiogenesis, breast cancer development and progression were not associated with lymphangiogenesis [31].

Another pathogenesis study by Versmold et al. showed that the underexpression or transcriptional silencing via the methylation of CPG island II of the *PROX1* gene might be responsible for breast cancer progression and its spread via blood vessels. More specifically, increased methylated *PROX1* alleles were found in 52% and 57% of primaries and brain metastases, respectively. Additionally, PROX1 protein expression was significantly reduced in brain metastases and breast cancer tissues [32]. Table 2 summarizes the main findings of studies reporting on the expression and role of PROX1 in breast cancer.

### 2.6. Esophageal Cancer

Given that both angiogenesis and lymphangiogenesis play a concerted role in solid tumor growth and spread, Loges et al. studied the formation of new blood and lymph vessels in primary esophageal carcinomas. A statistically significant relationship was found between all three lymphoendothelial markers when measured in surgical samples of esophageal carcinoma; LYVE with PROX1 and VEGFR-3, and VEGFR-3 with PROX1. However, the expression of all these lymphoendothelial cell antigens was not correlated to the LN status [33].

According to Yokobori et al., PROX1 is a negative prognostic factor in esophageal carcinoma. More specifically, PROX1 expression was found to be higher in esophageal squamous cell carcinoma (ESCC) tissues, compared to normal tissues. Patients with higher PROX1 expression also had an increased nuclear accumulation of HIF1α and more advanced lymphatic and hematogenous spread. High PROX1 and HIF1α expression were also correlated with low levels of the epithelial cell marker, E-cadherin. Analysis of overall and cancer-specific survival indicated that elevated PROX1 expression in ESCC was significantly correlated with poor prognosis. Inhibiting the actions of PROX1 or silencing the *PROX1* gene could represent a novel therapeutic strategy in ESCC treatment [34].

### 2.7. Gastric Cancer

The role of PROX1 in gastric cancer has been the subject of investigation in four studies, which, interestingly, lead to conflicting conclusions.

Taban et al. examined the expression of PROX1 in gastric cancer surgical samples and found that the vast majority of examined specimens were positive for PROX1. PROX1 protein and gene expression were detected with different methods, namely, IHC and RNAscope, an in situ hybridization method that detects mRNA PROX1 amplification. High amplification in the PROX1 mRNA score correlated significantly to LN metastasis and tumor grade. No association was observed between PROX1 expression and histopathology, tumor size, or the presence of distant metastasis. Interestingly, perineural and lymphatic invasion areas showed intense PROX1 staining and a high mRNA PROX1 amplification rate, implying an active role for PROX1 in tumor spread [35]. Park et al. found increased PROX1 expression in gastric cancer samples and metastatic LNs, in comparison with normal gastric mucosa and non-metastatic LN tissues. Patients with PROX1-positive tumors had significantly shorter OS and an elevated risk of death [36]. The correlation of PROX1 overexpression with poorer prognosis in cases of gastric cancer is also reported in the study conducted by Ueta et al. Patients with high PROX1 expression had significantly lower 5-year OS and a lower recurrence-free survival rate compared to their low-PROX1-expressing counterparts. Additionally, patients with high PROX1 expression had a worse prognosis according to cancer stage (pStage: I–II vs. III–IV), N status (pN: N0 vs. N1-3), lymphatic vascular invasion (negative vs. positive), and vascular invasion (negative vs. positive). Even though the mechanisms underlying worse prognoses remain unknown, the authors suggested that in the future, suppressing PROX1 or its downstream partners could be an effective therapeutic strategy [37].

Conversely, Laitinen et al. reported that patients with gastric cancer and high PROX1 expression had significantly prolonged 5-year cancer-specific survival. Moreover, PROX1 positivity correlated with better prognoses in the following subgroups: males, patients aged <66 years, patients with an intestinal cancer type, and patients with a tumor size of <5 cm. After multivariable analysis, PROX1 proved to be an independent prognostic marker for better prognosis [38].

According to recent data, the dysregulation of micro RNAs (miRs), which represent non-coding RNAs with a critical role in the regulation of the expression of tumor-suppressor genes and oncogenes, contributes to gastric cancer development. In the study by Zhang et al., microRNA-489 (miR-489) was found to be downregulated in gastric cancer tissues and correlated negatively with PROX1 protein expression. Experimental findings showed that *PROX1* is a target gene of miR-489. Consequently, the downregulation of miR-489 is linked to the overexpression of PROX1 and tumor spread, while conversely, miR-489 overexpression suppresses PROX1 expression and mitigates tumor proliferation and invasion. In that manner, reducing PROX1 expression via the *PROX1* gene regulator, miR-489, could constitute a new therapeutic approach [39].

### 2.8. Colon Cancer

In normal colonic tissue, PROX1 is usually present in vascular lymphatic endothelial as well as neuroendocrine epithelial cells. There is evidence that, during colonic carcinogenesis, the Wnt/beta-catenin pathway signaling is upregulated, affecting the activity of several target genes, such as *PROX1*.

Apart from the lymphatics, PROX1 is highly expressed in colorectal cancer tissue compared to background tissue samples. It should be highlighted that tumors with local invasion are characterized by higher expression levels [40]. Positive staining is also observed in colon adenoma samples, whereas the highest PROX1 levels are seen in the severe dysplastic areas. Conversely, staining is absent or is only rare in normal colonic epithelium samples or in hyperplastic polyps [41,42].

Skog et al. correlated the expression of PROX1 in colonic cancer samples with clinicopathological characteristics. Of the tumor samples, 91% were PROX1-positive. High PROX1 expression was significantly associated with high-grade tumors and worse 5-year colorectal cancer-specific survival (CSS). Moreover, female colon cancer patients with high PROX1 expression exhibited an unfavorable CSS. However, in cases of rectal cancer, specifically, no correlation with survival was reported [42].

According to the findings of Lu et al., the overexpression of PROX1 correlated with E-cadherin downregulation, an advanced tumor stage, and LN metastasis. Interestingly, PROX1 inhibits the expression of E-cadherin, in this way promoting epithelial-mesenchymal transformation (EMT) and, thus, colon cancer progression and invasiveness [43]. In line with this study, another group of researchers reported that in cases of colon cancer, PROX1 positivity correlated significantly with tumor size, histologic type, lymphovascular invasion, cancer stage, depth of invasion, and LN metastasis. Moreover, patients with higher PROX1 expression had an elevated risk of death and reduced OS rates after adjustments were made for age, sex, and tumor size [44]. Similar results are published by Abdelrahman et al. in a cohort of stage-II/III colon cancer cases [45].

In contrast to the aforementioned studies, Zhang et al. reported that high PROX1 expression in colonic tumor tissues, as well as in paracancerous tissues, did not correlate with the LN status or N-staging [46].

As previously mentioned, PROX1 is typically co-expressed in the neuroendocrine cells of the gastrointestinal tract. Its presence is more prevalent in enteroendocrine cells expressing hormones such as peptide YY (PYY), cholecystokinin (CCK), and glucagon-like peptide-1 (GLP-1) [41]. Jernman et al. evaluated the nuclear expression of PROX1in primary rectal neuroendocrine tumors (NET) and the associated metastases. Expression levels correlated with metastatic potential, tumor size, WHO grade, and poor patient survival. However, in cases of known metastasis, no correlation between PROX1 expression and progression-free or disease-specific survival could be identified [47].

### 2.9. Hepatobiliary Cancer

Carreira et al. studied the lymphatic distribution in cases of hepatocellular carcinoma (HCC), irrespective of the presence or absence of underlying cirrhotic disease, as well as in cases of liver metastases. According to the staining results for PROX1 expression, lymphatics were visible only in the tumor capsule and in the surrounding liver but were not visible within the tumor or between the nodules, in either HCC or in liver metastases. The mechanism underlying this intratumoral lymphatic absence remains unknown and raises questions about its influence on the tumor microenvironment and growth [48].

Further data from studies that incorporated HCC patients’ tissues indicated that higher PROX1 levels correlated with well-differentiated tumors, while lower expression levels correlated with poorly differentiated ones. Furthermore, patients with high PROX1 expression had a significantly higher 5-year OS rate; however, no significant difference in the DFS between the two groups was observed. Concomitant genetic analyses of the *PROX1* gene performed in cases of HCC showed no mutations in the coding region of *PROX1* or any loss of heterozygosity (LOH). The authors of that study stated that the mechanism that regulates the *PROX1* gene remains unknown [49].

The role of PROX1in the carcinomas of the biliary tract has been also studied. PROX1 staining was intense in the normal gallbladder epithelium and in normal intra- and extra-hepatic bile duct epithelial cells but varied in intensity in carcinomas of the biliary system. The PROX1 protein was absent or reduced in the majority of the studied carcinomas. This reduction did not correlate with clinicopathological variables. Further genetic analysis of *PROX1* demonstrated the presence of epigenetic silencing and genomic deletions. These genetic alterations, which are widely considered to be responsible for the inactivation of tumor-suppressor genes, could explain the strongly reduced levels of PROX1 protein in carcinomas of the biliary tract [50].

### 2.10. Pancreatic Cancer

PROX1 is found to be slightly reduced in pancreatic cancer tissue. in contrast to its abundant expression in a normal pancreas. Patients with reduced survival (less than 6 months) show a significantly lower *PROX1* gene expression compared to those with longer OS [51].

Similarly, Saukkonen et al. demonstrated that the combined high expression of PROX1 and β-catenin was significantly correlated with a longer CSS and a lower risk of death from pancreatic ductal adenocarcinoma [51,52].

The main characteristics and findings of studies that evaluated the expression and clinical significance of PROX1 in gastrointestinal and hepato-biliary-pancreatic cancer are presented in Table 3.

### 2.11. Soft Tissue Tumors

PROX1 has been found to be predominantly expressed in vascular endothelial neoplasms. More specifically, PROX1was observed to be present in lymphangiomas and hemangiomas (especially in venous and spindle-cell hemangiomas), whereas it was reported as absent in capillary and cavernous hemangiomas. Furthermore, the vast majority of Kaposi sarcomas seem to be PROX1-positive, while its expression in cases of angiosarcoma reaches 50%. Non-endothelial mesenchymal tumors exhibit the lowest percentage of PROX1 expression [53].

In angiosarcomas (primary or secondary), certain morphological features characterizing lymphatic differentiation, such as a hobnail cell appearance and kaposiform architecture, correlate with immunopositivity for D2-40, PROX1, and VEGFR-3. However, the correlation between PROX1 and such characteristics does not seem to be statistically significant. On the other hand, cases with lymphangiosarcomatic differentiation showed positivity for D2-40 and PROX1. Since no difference is observed in clinical outcomes between this group and the rest of the tumors, the clinical utility of separating these from other angiosarcomas remains rather unclear [54].

Hadj-Hamou et al. aimed to find genome and transcriptome abnormalities that could characterize and differentiate radiation-induced breast angiosarcomas from primary ones. They observed, specifically, in radiation-induced breast angiosarcomas a concrete deregulation in the marker genes of the lymphatic endothelial cells, such as podoplanin (PDPN), PROX1, VEGFR3, and endothelin receptor A (EDNRA), which was probably induced through chronic oxidative stress. This unique transcriptome signature could differentiate angiosarcomas, depending on their etiology [55].

### 2.12. Renal Cell Carcinoma (RCC)

According to the existing data, RCCs are characterized by limited lymphangiogenesis, with a concomitant reduced the intratumoral expression of VEGF-D and PROX1; the expression levels of lymphangiogenic factors are significantly lower in RCC tissues compared to normal control tissues. This fact could explain the low nodal metastatic rate of clear-cell RCC, in contrast to its well-known predominant hematogenous spread [56].

### 2.13. Bladder Carcinoma

There is little evidence regarding lymphangiogenesis in urothelial carcinoma. Bolenz et al. focused on lymphatic vessel density (LVD) and the presence of lymphangiogenesis in urothelial carcinoma of the bladder. LVD was measured in the intratumoral (ITLVD), peritumoral (PTLVD), and non-tumoral regions (NTLVD). The confirmation of lymphatic origin was made by using several specific lymphatic markers, such as PROX1, LYVE-1, and VEGFR-3. PTLVD was reported to be higher than ITLVD and NTLVD in all tumor stages. Furthermore, an elevated PTLVD was significantly associated with the presence of concomitant carcinoma in situ (CIS). A higher ITLVD was significantly associated with a higher pT stage, higher histological grade, and sessile tumor architecture [57].

### 2.14. Gynecological Cancer

In cases of invasive squamous cell carcinoma of the uterine cervix, LVD, as assessed by the lymphatic markers D2-40, LYVE-1, and PROX1, did not correlate with LN metastasis, FIGO stage, or tumor grade. Interestingly, PROX1 ITLVD and PROX1 and LYVE-1 PTLVD correlated significantly with the degree of stromal inflammation, indicating that inflammation may play a role in cervical cancer-associated lymphangiogenesis. However, the expression of PROX1 in the tumor cells did not correlate significantly with any of the clinicopathological parameters studied; therefore, this study could not support that PROX1 has any prognostic value in cervical cancer [58].

In contrast to the abovementioned findings, Cai et al. supported that in cervical cancer, a higher LVD was significantly associated with an advanced FIGO stage, poorer cell differentiation, and pelvic lymphatic nodal metastasis. Lymph vessel density proved to be an independent predictor of poor patient outcomes in squamous cervical cancer. Moreover, in vitro cultures showed that tumor-associated lymphatic endothelial cells from cervical cancer tissues do actively promote lymphatic metastasis. PROX1 served as a diagnostic marker for tumor-associated lymphatic endothelial cells [59].

**Figure 2:**
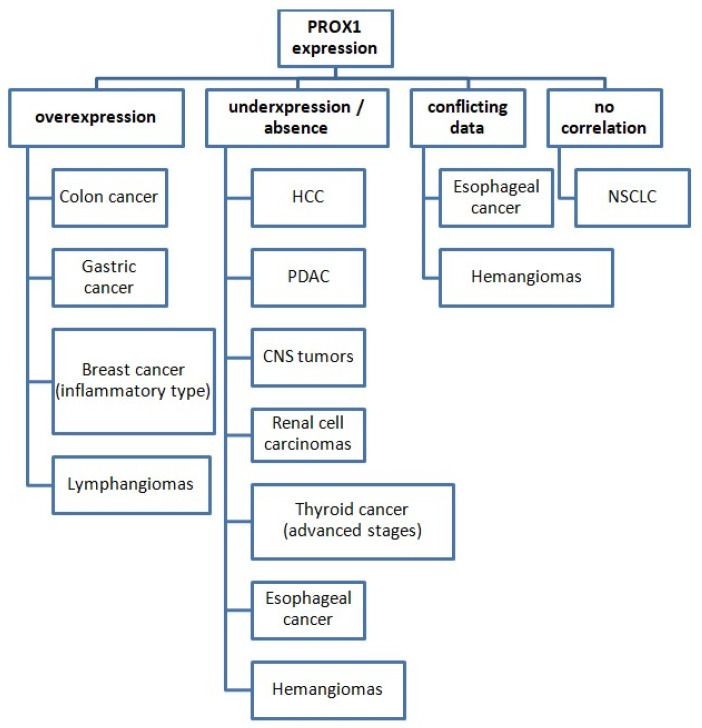
PROX1 expression status in various cancer types (compared with normal tissues). Abbreviations: HCC: hepatocellular carcinoma, PDAC: pancreatic ductal adenocarcinoma, CNS: central nervous system, NSCLC: non-small-cell lung cancer.

The identification and quantification of PROX1 expression have served several different purposes in the aforementioned studies concerning various cancer types. Its expression pattern among various malignant tumors is presented in Figure 2.

PROX1 has been used as a lymph-specific marker during the histological examination of cancer tissues and has been also studied at the molecular level to investigate its role in tumorigenesis. Additionally, several studies examined the prognostic significance of PROX1 as well as its suitability as a new therapeutic target.

## 3. The Role of PROX1 in Pathogenesis, Diagnosis, Prognosis, and Therapy of Cancer

### 3.1. PROX1 as a Lymph-Specific Marker

The study of the lymphatic vessel system of the tumor and of the affected LNs is essential for understanding the tumor’s behavior. PROX1 has been used as a lymph-specific marker to identify and quantify the lymphatic vessels and investigate tumor and lymph node lymphangiogenesis. More specifically, nowadays, the management of several tumors depends on the identification of SLNs, since this denotes regional LN spread. Two studies, one incorporating OSCC tissues and one concerning BC patient tissues, reported high PROX1 levels, along with other lymphatic-specific markers, such as LYVE-1 and podoplanin, in SLNs compared to control LNs, proving that active lymphangiogenesis does occur in the SLNs before metastasis. Interestingly, the levels of the lymphatic markers in SLNs were even higher when the tumor was VEGF-C-positive, suggesting an active role of VEGF-C in lymphangiogenesis [19,22]. Several studies used PROX1 in order to quantify intra-and peritumoral lymphangiogenesis, mainly expressed as LVD, and further correlated this to clinicopathological variables and cancer prognosis [28,29,30].

### 3.2. PROX1 as a Diagnostic and Histological Marker

Apart from staining the lymphatic tissues, PROX1 expression is also noted in tumor cells, with a nuclear or cytoplasmatic distribution, and in the tumor microenvironment. In astrocytic gliomas, a higher expression of PROX1 is found in all tumor grades compared to non-neoplastic lesions; higher expression correlates with an advanced tumor grade (III, IV), suggesting PROX1 as a new molecular tool in tumor grading and in histological discrimination between benign and malignant lesions [18,19]. In gastric cancer, there is also a positive correlation between PROX1 expression and tumor grade. PROX1 staining is also more intense in the areas of the invasion front, indicating PROX1 as a new tumor marker for gastric cancer [33]. In pancreatic cancer, the expression of PROX1 is downregulated, while, conversely, PROX1is abundant in the normal exocrine pancreas. The IHC staining of PROX1 could help in the future with the differentiation of pancreatic carcinomas [49]. Researchers point out that PROX1 could serve as a helpful diagnostic marker during the histologic examination of vascular endothelial tumors. Lymphangiomas and hemangiomas (mainly venous and spindle-cell subtype), as well as the vast majority of Kaposi sarcomas and reti- and kaposiform hemangioendotheliomas, are associated with the overexpression of PROX1. Interestingly, radiation-induced breast angiosarcomas have their own transcriptome signature, with a panel of deregulated marker lymphatic genes such as PROX1, podoplanin, VEGFR3, and EDNRA, which allows its discrimination from primary breast angiosarcomas [53].

### 3.3. PROX1 in the Pathogenesis and Therapy of Cancer

There is evidence that PROX1 may be associated with both the progression and even the suppression of tumorigenesis, but this is highly dependent upon the specific type of cancer. Several researchers have conducted in vitro and animal studies to elucidate the molecular pathways and mechanisms underlying PROX1 multifactorial activity in cancer. In thyroid cancer, PROX1 is downregulated; this is linked to more aggressive tumor behavior and tumor progression. The molecular pathways involved in PROX1 inactivation are complex. One possible mechanism could be the activation and dysregulation of the NOTCH signal pathway [22,23]. The restoration of PROX1 activity could represent a possible novel therapeutic intervention.

In breast cancer, lymphangiogenesis plays a major role in metastasis. The levels of Interleukin 24 (IL-24), a cytokine that inhibits tumor progression, and its receptor, the IL-24 receptor (IL-24R), are found to be reduced in breast cancer samples and are associated with increased levels of lymph-specific markers, such as podoplanin, PROX1, and LYVE-1. Targeting the elevation of IL-24 could probably inhibit lymphangiogenesis and limit the tumor’s aggressiveness [27]. Especially in the case of inflammatory breast carcinoma, active angiogenesis and lymphangiogenesis are evident through the upregulation of angiogenic growth factors and lymphangiogenesis-related genes. Inhibiting both pathways could offer an effective therapeutic strategy for breast cancer [26]. Interestingly, the dysregulation of PROX1, along with PDPN, VEGFR3, and EDΝRA, seems to play a pathogenetic role in the development of post-radiotherapy breast angiosarcomas, which, through the identification of those specific genes, present a discriminating genetic “signature” [53]. The upregulation of PROX1 is found in the aforementioned studies, in relation to intense lymphangiogenesis.

However, another study found a reduced expression of PROX1 in breast cancer tissues and in brain metastases from breast cancer patients, compared to normal breast tissue. Genomic analysis revealed the hypermethylation of PROX1 and the subsequent transcriptional silencing of *PROX1*. Assuming that in breast cancer, *PROX1* acts as a tumor-suppressive gene, the demethylation of *PROX1* and reactivation could represent a possible therapeutic strategy [30].

Similarly, in the carcinomas of the biliary tract, promoter *PROX1* hypermethylation, and genomic deletions lead to the inactivation of *PROX1* and reduced protein level and contribute in that manner to biliary tract carcinoma development. Inhibiting these molecular pathways could suppress tumor expansion [48].

Ιn ESCC, *PROX1* seems to act as a tumor-promoting gene, since high levels were linked to a poor prognosis [32]. At the molecular level, high PROX1 expression levels were combined with high levels of HIF-1a, a PROX1 regulator, and low levels of E-cadherin, suggesting that PROX1 induces EMT and metastasis via HIF-a. The silencing of PROX1 could represent a therapy option for ESCC patients. Similarly, in gastric cancer, elevated PROX1 expression levels were related to poor prognosis and to a high proliferation rate (assessed with the Ki-67 labeling index), and increased lymphatic vessel density [34,35]. Moreover, IHC analysis reveals that the expression of PROX1 is more intense in the perineural infiltration areas and in tumor emboli than in the tumor itself [33]. Altogether, these data suggest that PROX1 promotes lymphangiogenesis, proliferation, and gastric cancer development. The knockdown of *PROX1* could reverse these events and ameliorate patients’ prognosis. In the study conducted by Zhang et al., miR-489 was significantly downregulated in gastric cancer tissues and was correlated to negative PROX1 protein expression. PROX1 expression could be enhanced via miR-489 downregulation. On the other hand, focusing on miR-489 re-expression could inhibit the deleterious effects of PROX1 in tumor expansion in gastric cancer [37].

In colon cancer, the activation of the Wnt signaling pathway is actually believed to mediate the self-renewal and proliferation of colon cancer cells, contributing to tumor relapse and metastasis. The Wnt/β-catenin pathway leads to the cytoplasmatic accumulation of β-catenin. Through its further intranuclear translocation, β-catenin acts as a transcriptional co-activator of certain transcription factors that belong to the TCF/LEF family. The adenomatous polyposis coli (APC)/β-catenin/T-cell factor (TCF) pathway plays a significant role in the initiation of CRC cancer. Mutations of APC, as seen in familial adenomatous polyposis, block β-catenin degradation and result in hundreds of polyps that progress in CRC at an early age. Experiments on mice have shown that PROX1 is a specific target of the β-catenin/TCF pathway, playing an essential role in the transition from benign colon adenoma to carcinoma in situ by affecting cell adhesion and polarity.

Pathological studies in colon adenomas and adenocarcinomas revealed high PROX1 expression in the majority of the cases, whereas, in the areas of colon adenomas with severe dysplasia, high PROX1 levels were combined with accumulated β-catenin protein, implicating an activated Wnt signaling pathway. PROX1 seems to play an important role in the transition to a more malignant phenotype [39]. In colon cancer, high PROX1 levels are linked to low E-cadherin levels, and both are associated with LN metastasis and Duke’s stage. PROX1 seems to downregulate E-cadherin and, thus, promotes EMT [42].

### 3.4. PROX1 as a Prognostic Marker in Cancer

**Figure 3:**
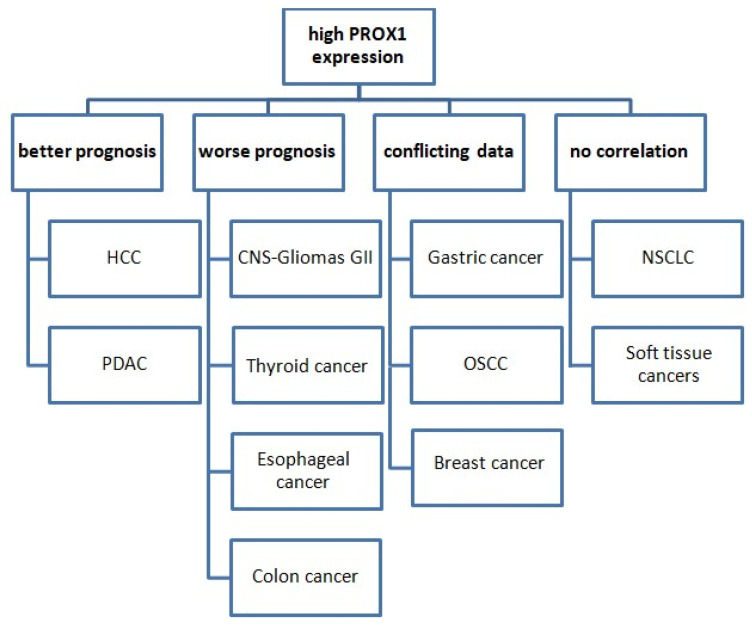
The prognostic value of PROX1 expression in various cancer types. Abbreviations: HCC: hepatocellular carcinoma, PDAC: pancreatic ductal adenocarcinoma, CNS: central nervous system, OSCC: oral squamous cell carcinoma, NSCLC: non-small-cell lung cancer.

It is noteworthy that the prognostic value of PROX1 expression varies among various types of cancer (Figure 3).

In more detail, PROX1’s high expression is associated with worse prognoses and shorter OS in cases of CNS tumors, colon, gastric, and esophageal cancer. Conversely, PROX1 overexpression could serve as a prognostic marker of better prognosis and prolonged survival in cases of pancreatic and HCC. Moreover, the existing data are conflicting in terms of the prognostic value of PROX1 in breast cancer and OSCC, while no corresponding reports are available for lung cancer. There are no studies examining PROX1 as a prognostic marker in soft-tissue tumors. In terms of lung cancer, data are very limited. PROX1 has, so far, shown no prognostic significance in NSCLC.

## 4. Conclusions

The scientific community has shifted its focus to elucidating the molecular signature behind tumor pathogenesis. Understanding those complex molecular mechanisms is of the utmost significance for the development of novel, personalized therapeutic strategies that will greatly benefit patients’ prognosis. The contribution of neoangiogenesis to various tumors’ carcinogenesis and progression has been extensively studied, with many new agents that are targeting this specific procedure currently being developed and tested. Research on the effects of lymphangiogenesis in cancer pathogenesis remains limited in comparison. However, molecules driving or suppressing lymphangiogenesis seem to play key roles in tumorigenesis, heavily impacting further disease progression. Among them, PROX1 has exhibited important tumor-suppressing as well as tumor-enhancing properties, depending on the tumor’s organ of origin, according to the data reported by a plethora of studies. The up- or downregulation of PROX1 has been associated significantly with a multitude of clinicopathological parameters, including patients’ survival. More research is needed to shed further light on the tumor-promoting and tumor-suppressing processes in which PROX1 is implicated as blocking PROX1 or enhancing its expression, depending on the type of tumor, could represent a promising therapeutic strategy for the management of many aggressive malignancies. However, these interventions could impact lymphangiogenesis in normal body tissues; therefore, the development of such agents should be accompanied by extensive testing to explore any possible side effects. We believe that research unveiling the therapeutic potential of targeting lymphangiogenesis and angiogenesis is of the utmost importance for the improvement of tumor patients’ disease management.

## Figures and Tables

**Table 1:** PROX1 expression in CNS, oral squamous cell and thyroid cancer tissues, and clinicopathological associations.

Tissues	Methods	Results	Ref.
20 PCNSL samples 8 DLBCLsamples 20 GBM samples	IHC	PROX1 expression (in LEC) absent in PCNSL and GBM tissues present in DLBCL tissues (62.5%) associated with the presence of lymph vessels	[16]
56 grade I–IV astrocytic glioma samples	IHC	PROX1 expression (in CC) increased with grade progression positively correlated with glioma tumor grade (*p* < 0.001)	[18]
116 grade II glioma samples	IHC	PROX1 expression is mainly low in investigated tissues (in CC) high PROX1 expression (>10% cells) is associated with a shorter OS (*p* = 0.018) PROX1 is an independent marker for OS in grade-II gliomas	[19]
23 metastasis-free SLNs of 10 OSCC **Controls:** 10 LNs	RT-PCR	Positive correlation between the expression levels of the lymphatic markers (PROX1, VEGFR-3 and LYVE-1) in SLNs from OSCC patients (in SLNs) no difference in VEGFR-2 and PROX-1 levels to controls, only LYVE-1 higher than controls (*p* < 0.01)	[20]
163 OSCC samples **Controls:** 5 normal oral mucosa specimens	IHC RT-PCR	PROX1 expression (in CC-nuclear) higher in OSCC (especially if LN+) associated with T, LN+, cTNM, and LVD (*p* = 0.0023, <0.0001, <0.0001 and <0.0001, respectively) associated with worse prognosis (*p* < 0.0001) is a prognostic factor for DFS (*p* = 0.0039)	[21]
40 OSCC samples **Controls:** non-tumor margins	IHC RT-PCR	PROX1 gene and protein expression (in CC) lower in OSCC tissues compared to normal oral mucosa not associated with age, tumor location, classification, grade, pTNM, LI, PNI, local recurrence Patients with positive PROX1 amplification had better prognosis and OS compared to patients with negative amplification (*p* = 0.08, log-rank)	[22]
97 TC samples **Controls: **4 normal thyroid tissues	IHC Western blot	PROX1 expression downregulated in TC tissues (in CC)	[23]
11 FTC samples **Controls:** 11 normal thyroid samples	RT-PCR	PROX1 expression (in CC) higher in stages I–II compared to more advanced stages (III–IV) of TC associated with reduced survival (*p* = 0.00067)	[25]

Abbreviations: CNS: central nervous system, PCNSL: primary central nervous system lymphoma, DLBCL: diffuse large cell B-lymphoma, IHC: immunochemistry, LEC: lymphatic endothelial cells, CC: cancer cells, OSCC: oral squamous cell carcinoma, p: pathological, LI: lymphatic invasion, PNI: perineural invasion, DFS: disease-free survival, TC: thyroid cancer, FTC: follicular TC, GBM: glioblastoma, RT-PCR: Reverse transcription-polymerase chain reaction, LYVE-1: lymphatic vessel endothelial hyaluronan receptor, SLN: sentinel lymph node, LN: lymph nodes, OS: overall survival, pn: perineural invasion, FTC: follicular thyroid cancer.

**Table 2:** Synopsis of studies evaluating the expression and role of PROX-1 in breast cancer.

Tissues	Methods	Results	Refs.
16 Inflammatory and 20 non-inflammatory BC specimens (non-stage-matched)	real-time RT-PCR	PROX1 expression (in LEC) Higher mRNA PROX1levels in inflammatory BC (*p* = 0.005) Higher PROX1 expression and higher LECP in inflammatory lesions (*p* < 0.005)	[28]
127 BC samples	IHC	High PROX-1 expression (in CC) inversely correlated with IL-24 expression (insignificantly)	[29]
63 SLNs from T1N0-T2N0 BC patients **Controls:** 30 LNs	real-time RT-PCR	PROX-1 expression (in SLNs) higher in SLNs compared with control LNs (*p* < 0.05) correlated with high tumor VEGF-C expression in SLNs (*p* < 0.05)	[30]
32 in situ ductal BC samples, 55 BC samples **Controls:** 23 normal breast samples, 7 fibrocystic lesion samples	IHC	PROX-1 expression (in LEC), expressed in lymph vessels but not in blood vessels Minimal or absent lymph vessel proliferation in tumor tissues	[31]
33 primary BC samples **Controls:** paired normal breast tissues	real-time RT-PCR	Reduced PROX1 expression in brain metastases (in CC) Increased PROX1 gene methylation in primary tumor tissues (especially ER+ tissues) and brain metastases No association between PROX1 methylation and age, PR/HER2/LN status, tumor grade, or size	[32]

Abbreviations: BC: breast cancer, IHC: immunochemistry, RT-PCR: reverse transcription polymerase chain reaction, LYVE-1: lymphatic vessel endothelial hyaluronan receptor, VEGF: vascular endothelial growth factor, SLN: sentinel lymph node, LN: lymph nodes, vs: versus, LVD: lymphatic vessel density, ER: estrogen receptor, PR: progesterone receptor, OS: overall survival, LEC: lymphatic endothelial cells, CC: cancer cells, LECP: lymphatic endothelial cell proliferation.

**Table 3:** Synopsis of studies evaluating the expression of PROX-1 in gastrointestinal and hepato-biliary-pancreatic cancer.

Tissues	Methods	Results	Refs
28 colon cancer samples **Controls:** 28 normal colon samples	RT-PCR	PROX1 expression (DNA extracted from CC) Increased in cancer tissues Positively associated with local invasion (p N/A)	[40]
241 human cancer samples **Controls:** 241 paired normal samples	Gene profiling array: PROX-1 mRNA	PROX1 expression in CC higher in severely dysplastic and CRC tissues lower in breast, uterine, lung, kidney, ovarian, and thyroid tumor tissues exhibited co-positivity for β-catenin and correlated with low levels of p53	[41]
517 colon cancer samples	IHC	PROX1 expression in CC ○ higher in grade 3-4 compared to grade 1-2 tumors (*p* < 0.0001) ○ positively associated with worse 5-year CCSS (*p* = 0.0045) ○ not correlated with age, tumor location, gender, or Dukes stage PROX1 is not an independent prognostic factor (multivariate survival analysis)	[42]
136 colon cancer samples	IHC	PROX1 expression (in CC) correlated with reduced E-cadherin expression (*p* = 0.0005) regional LN infiltration (*p* = 0.000009) Dukes Stage (*p* = 0.005)	[43]
528 colon cancer samples **Controls:** 528 paired normal colon samples	IHC	PROX1 expression higher in cancer tissues (in CC) PROX1 (+) tumor patients exhibited shorter OS (*p* < 0.001) PROX1 expression associated with ○ tumor size (*p* = 0.020) ○ T stage (T1/T2 vs T3/T4, *p* = 0.001) ○ LN infiltration (*p* = 0.022) ○ M1 (*p* = 0.187) ○ L1 (*p* = 0.001) ○ Pn1 (*p* = 0.196)	[44]
60 cases of colon cancer stages II to III—samples	IHC	PROX1 expression (in CC) associated with left-sided tumors (*p* = 0.022) high grade tumors (*p* = 0.004) lymph node metastasis (*p* = 0.009) advanced tumor stage (*p* = 0.016) shorter DFS (*p* = 0.010) shorter OS (*p* = 0.023)	[45]
47 colon cancer samples **Controls:** 47 paracancerous tissue samples	quantitative RT-PCR	High PROX1 expression in both cancer (in CC) and paracancerous tissues without significant differences	[46]
50 gastric cancer samples	IHC mRNA-scope	Gene amplification (in CC) correlated with ○ tumor grade (*p* = 0.05) ○ LN metastasis (*p* = 0.033) No correlation between PROX1 and histopathology, tumor size or metastases	[35]
327 gastric cancer samples	IHC	PROX1 overexpression in primary tumors and LN metastases (in CC and LNs) PROX1 expression associated with ○ age (<cut-off of 58.7 y; *p* < 0.001) ○ differentiation (*p* = 0.001) ○ advanced tumor stage (*p* = 0.012) ○ LN metastases (*p* = 0.002) ○ worse OS (*p* < 0.001) ○ elevated risk of death (HR = 1.662, 95% CI = 1.180–2.339)	[36]
99 gastric cancer samples	IHC	PROX1 expression (in CC) associated with advanced pathological Stage (*p* = 0.02) LN metastasis (*p* = 0.0002) Lymphatic vascular invasion (*p* = 0.03) Vascular invasion (*p* = 0.007) shorter 5-y OS (*p* < 0.001) shorter recurrence free survival (*p* < 0.001)	[37]
283 gastric cancer samples	IHC	PROX1 positivity (in CC) associated with ○ better prognosis in males (*p* = 0.019) ○ <66 y (*p* = 0.007) ○ intestinal type (*p* = 0.025) ○ tumor size <5 cm (*p* = 0.030) High PROX1 expression correlated with ○ better 5-y CSS (*p* = 0.004) ○ inversely with diffuse cancer type (*p* = 0.002)	[38]
54 esophageal carcinoma samples	quantitative RT-PCR MVD assessment	PROX1 expression in CC and LEC No correlation between PROX-1, LYVE or VEGF-3 expression and MVD No correlation between the endothelial LVD and LN status	[33]
117 esophageal cancer samples	IHC	PROX1 overexpression in esophageal cancer tissues (in CC) PROX1 overexpression associated with ○ LN infiltration (*p* = 0.09) ○ Presence of metastasis (*p* = 0.04) ○ increased HIF1a nuclear accumulation (*p* = 0.004) High PROX1 expression in ESCC is an independent prognostic marker of poor survival (*p* = 0.0064)	[34]
25 HCC samples, 17 liver metastases samples (from the GI tract, pancreas, and ovary cancers)	IHC	PROX1 expression in LEC lymphatics restricted to the tumor margin and surrounding liver in HCC and liver metastases lymphatic distribution may impair molecular and cellular transport in these tumors	[48]
52 HCC samples **Controls:** 52 non-cancerous liver tissue samples	Semiquantitative RT-PCR IHC	PROX1 overexpression (in CC) correlated with high tumor differentiation lower tumor stage better 5-y OS (*p* = 0.014)	[49]
36 PDAC samples **Controls:** 30 normal pancreatic tissues	quantitative RT-PCR IHC	PROX1 expression in both CC and LEC Mean PROX1 gene expression lower in patients with survival <6 months vs patients with longer survival. Correlation of PROX1 expression in PDAC with longer survival (borderline significance, *p*=0.086)	[51]
156 PDAC samples	IHC	PROX1 expression in CC High PROX1 expression associated with ○ older patients (*p* = 0.038) ○ lower risk of death from PDAC in multivariate analysis (HR = 0.63, *p* = 0.026) High PROX-1 expression is an independent prognostic marker for better survival	[52]

Abbreviations: HCC: hepatocellular carcinoma, ESCC: esophageal squamous cell carcinoma, IHC: immunochemistry, RT-PCR: reverse transcription-polymerase chain reaction, LYVE-1: lymphatic vessel endothelial hyaluronan receptor, VEGFR: vascular endothelial growth factor, HIF1a: Hypoxia-inducible factor 1, 5-y CSS: 5 years of cancer-specific survival, SLN: sentinel lymph node, LEC: lymphatic endothelial cells, CC: cancer cells, LN: lymph nodes, LVD: lymphatic vessel density, MVD: micro-blood vessel density, OS: overall survival, DSF: disease-free survival, CSS: cancer-specific survival, PDAC: pancreatic ductal adenocarcinoma.

## Data Availability

Not applicable.
